# Safe and Time-Saving Method for Pharyngeal Reconstruction Using an Internal Mammary Artery Perforator Flap

**DOI:** 10.1055/a-2780-1488

**Published:** 2026-02-27

**Authors:** Cesar A. Gonzalez-Martinez, Yanko Castro-Govea, Brenda J. Tellez-Rodriguez, Karla V. Palomo-Barbosa, Mario A. Rico-Padilla, Oscar Vidal-Torres, Marco A. Treviño-Lozano, Cynthia M. Gonzalez-Cantu

**Affiliations:** 1 Plastic Surgery Department, University Hospital “Dr. José Eleuterio Gonzalez,” Autonomous University of Nuevo Leon, Monterrey, Nuevo Leon, Mexico; 2 General Surgery Department, University Hospital “Dr. José Eleuterio Gonzalez,” Autonomous University of Nuevo Leon, Monterrey, Nuevo Leon, Mexico

**Keywords:** internal mammary artery perforator flap, total laryngectomy, laryngeal cancer, head and neck oncological surgery, radiation

## Abstract

Head and neck squamous cell carcinoma often requires total laryngectomy (TL), creating complex defects, especially in irradiated patients. The internal mammary artery perforator (IMAP) flap is a promising reconstructive option, offering reliable vascularization with minimal donor site morbidity. However, postoperative characteristics such as retention/survival and complications in irradiated patients have not been fully addressed. A retrospective analysis was conducted on seven patients undergoing IMAP flap reconstruction after TL for advanced laryngeal carcinoma. All had prior radiotherapy. The procedures were designed using the Doppler ultrasound technique, and the second intercostal space IMAP was used in most patients, except for one. Flap elevation surgical time, defect coverage, complications, and hospital stay were evaluated. Our patients were 79 ± 4.3 years old. The average surgical time for flap elevation was 61 ± 5.34 minutes. The pharynx defect size was 4.57 ± 0.53 cm ^2^ , achieving full coverage in all cases. The mean hospital length of stay was 4.0 ± 1.52 days. One patient developed a postoperative hematoma; no flap necrosis or fistulas occurred after 3 months of follow-up. The flap survival rate was 100%. In this work, our IMAP flap design could be considered thin and elongated; however, due to the constant blood flow of the IMAP, we observed reliable results, and no partial or total necrosis was reported. Thus, the IMAP flap is a safe and effective alternative for laryngeal defect reconstruction in irradiated patients, proving excellent viability, favorable aesthetics, reduced morbidity, and minimal complications.

## Introduction

Squamous cell carcinoma of the head and neck is one of the greatest challenges in oncological surgery due to its aggressive behavior, high rate of local recurrence, and frequent treatment with chemotherapy and radiotherapy. 1 Survival rates, both overall and disease-free, have not shown significant improvements in patients with advanced disease. 2 Defects generated after surgeries such as total laryngectomy (TL) or laryngopharyngectomy involve critical structures, including the cervical esophagus and pharynx, requiring complex reconstructions to restore function and aesthetics. 3

Pharyngeal repair in patients with laryngeal cancer is especially complex due to the decreased vascularity caused by radiation used as a therapeutic method, leading to the rejection of free flaps, with pharyngocutaneous fistulas being the main complication. 3

The internal mammary artery perforator (IMAP) flap has proven to be a safe and versatile option for reconstruction in head and neck oncological surgery, 4 especially in cases where prior radiation therapy has compromised the quality of local tissues for its ability to provide reliable vascular coverage and minimal morbidity at the donor site. 5

The IMAP flap is an ideal choice for defects measuring up to 20 to 25% of cervical structures, including skin, mucosa, and soft tissues. 6 More extensive defects exceeding this percentage may require the combined use of free flaps, such as the anterolateral thigh (ALT) or radial flaps. 7

In previous studies, the overall complication rate associated with the use of the IMAP flap is reported to be low (5.6%). 7 8 Partial flap necrosis has been reported in 5%, while postoperative fistulas occur in 12% of the procedures. The flap survival rate is close to 95%, highlighting its reliability even in irradiated fields. 9 Additionally, it is less bulky, has shorter surgical times, and produces superior aesthetic results due to minimal morbidity at the donor site. 6

In this study, we decided to use a novel design for the laryngeal defect repair flap. This design features a narrow, elongated profile and is based on other flaps, such as the propeller flap used previously on the chest and neck, 10 which has been shown to have good survival due to high blood flow.

Furthermore, its effectiveness in patients with a history of radiation therapy in this area has not been evaluated. 1 This case series reports the experience of using the IMAP flap in seven patients with pharyngeal defects derived from oncological surgery. Complications, clinical evolution, and IMAP flap survival were evaluated up to 3 months postoperatively.

## Case

### Methods

#### Study Population—Case Selection

This study included patients over 18 years of age, regardless of sex, who underwent surgery in the years 2021 to 2023 by the Surgical Oncology Department for the treatment of squamous cell carcinoma of the larynx and who had previously received radiotherapy as a therapeutic method. The procedure was performed in the same intervention as the cancer resection.

Patients with conditions that could compromise the outcome of the surgery, such as coagulopathies, those on immunosuppressive treatment, pregnant women, and those with a history of cardiac surgery or surgery involving the internal mammary artery and hemodynamic instability, were excluded.

**Fig. 1:**
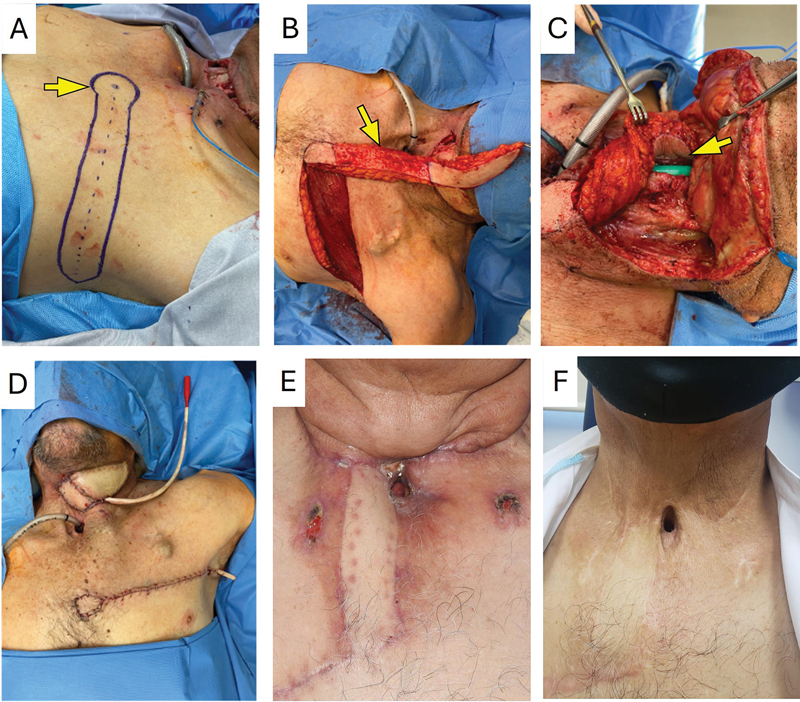
Design and execution sequence of the IMAP flap. ( **A** ) Circular marking (yellow arrow) over the IMAP flap and horizontal axial flap onto the medial axillary line. ( **B** ) Depithelialized flap (arrow) to pass it under the chest skin. ( **C** ) Partial fixation (arrow)of the flap over the defect. ( **D** ) Closure of the donating site. Flap under the chest skin and closure of the pharyngeal fistulae. ( **E** ) Surgical outcome follow-up at 2 years at 6 weeks. ( **F** ) Surgical outcome follow-up at 2 years. IMAP, internal mammary artery perforator.

**Fig. 2:**
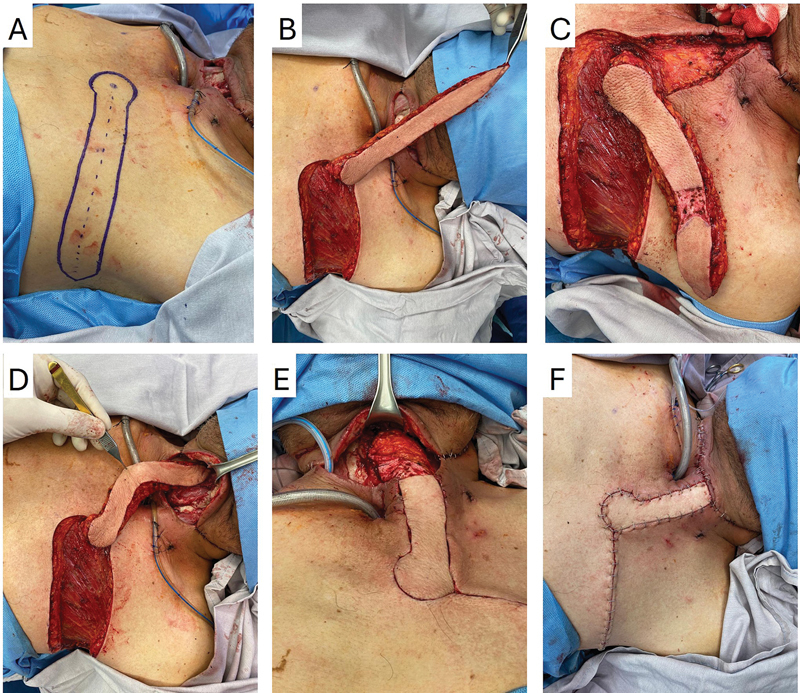
Design and execution sequence of the IMAP flap. ( **A** ) Circular marking (yellow arrow) over the IMAP flap and horizontal axial flap onto the medial axillary line. ( **B** ) Cephalic rotation of 100 degrees (arrow) to reach the neck. ( **C** ) De-epithelialized segment (arrow) for rotation and suture. ( **D** ) Suture of the distal portion of the flap over the pharyngeal defect. ( **E** ) Fixation of the flap after 180-degree rotation to use the distal portion of the flap as the anterior pharyngeal wall (arrow). ( **F** ) Closure of the flaps. IMAP, internal mammary artery perforator.

All study procedures were conducted in compliance with national and international regulations, including the Declaration of Helsinki. Our Institutional Review Board approved our project with the approval number: CP25-00006, and informed consent was obtained from all the patients, including the patient from whom the photographs were taken to form Figs. 1 and 2 .

#### Internal Mammary Artery Perforator Flap Surgical Technique

Preoperatively, the IMAP flap of the second intercostal space was located and marked using a Huntleigh Dopplex MD2 manual Doppler ultrasound to design the flap as a starting point. The flap was designed and marked following the horizontal axis of the chest until the anterior axillary line with a 2.5 to 3 cm width. This flap is performed with the arm open and with the aid of an auditory Doppler, which indicates the location of the perforator from which the procedure will be performed. Starting from this point, the design is literally horizontal toward the anterior axillary line, following the axis of the intercostal space, and ending slightly obliquely toward the armpit ( Figs. 1A and 2A ). The length depends on the distance needed for the coverage. The medial part of the flap is semicircular to allow rotation, and the lateral part is elliptic to allow skin closure.

First, the inferior edge of the designed flap is approached by incision to identify and dissect the perforator artery of the second intercostal space. Dissection is carried out keeping a suprafascial plane of the pectoralis major until the designed flap. In female cases, a fascia is located between the subcutaneous tissue and the breast tissue. We elevate the flap, including the fascia but not the breast tissue.

In some patients, two IMAP flaps can be found, and the one with the largest caliber is the best choice for the flap; the other one must be ligated. The IMAP flap perforator's course beyond its point of origin was followed through anatomic landmarks. This applies to all the propeller flaps, but if the perforators' arteries come from the same vertical axis, they can be preserved. The IMAP flap was dissected using blunt-tip dissection tweezers through the muscle fibers to identify any fixation points that raise twisting risk. Dissection was carried out from lateral to medial, keeping a suprafascial plane ( Figs. 1B and 2B, C ). To prevent compression of the pedicle, we ensured that the tunnel was wide enough, and the flap was evaluated for congestion after being tunneled.

The flap was rotated at 90 degrees in a cephalic direction to cover the defect on the neck. Two methods/pathways can be followed: Vertical incision to communicate the flap and the neck defect, or de-epithelialize the flap and make a subcutaneous tunnel towards the midline to pass the flap under the skin to cover the neck defect. Once in the neck, the flap was rotated 180 degrees on its axis, positioning the skin surface internally within the pharynx. Once again, the flap was marked with the zone that covers it, and the rest of the flap is de-epithelialized ( Figs. 1C and 2D, E ).

Subsequently, Vicryl 3–0 was used to attach the pharyngeal mucosa to the skin of the flap, and a hermetic test with saline solution and high pressure from the oral cavity to the pharynx was performed to confirm the correct closure of the stitches.

The chest incisions are closed with Monocryl 3–0 and skin staples, leaving a Blake 15fr drain probe for 2 to 3 days ( Figs. 1D and 2F ). Finally, at the end of the flap elevation, the dermis was bled at the most distal point to assess perfusion.

#### Postoperative Findings, Complications, and Follow-Up

For this study, clinical data were reported on patients who underwent IMAP flap surgery. Surgical data, including surgical time for flap elevation, size of pharyngeal defects, type of perforator used, hospital stay, and complications such as hematomas, fistulas, or infections, were also reported. Patients were followed up to 3 months after surgery.

### Results

This surgical technique was performed on seven patients (six males and one female) with an average age of 79 ± 4.32 years. All patients were diagnosed with advanced-stage squamous cell carcinoma of the larynx and previously treated with radiotherapy. The surgery was conducted in collaboration with a surgical oncologist, who performed laryngectomies as part of the treatment.

The average duration of the IMAP flap surgical technique was 61 ± 5.34 minutes.

The pharyngeal defect sizes were 4.57 ± 0.53 cm ^2^ , flap sizes ranged from 23 to 25 cm in length and from 2.5 to 3.0 cm in width, and a thickness of 12 to 25 mm. Complete coverage with the IMAP flap was achieved in all cases ( Table 1 ). The second perforator was the most frequently used (6/7 patients). The average hospital length of stay was 4.0 ± 1.52 days, and only one patient developed a hematoma associated with the intervention. A radial forearm flap was used to cover a large neck defect in one case and a fistula in the other.

**Table 1:** Demographic and clinical characteristics of the internal mammary artery perforator flap cases

Case	Sex	Age	Defect size (cm ^2^ )	Flap size (W × L cm)	IMAP flap perforator	Flap elevation surgical time (minutes)	Complications	Hospital stay (days)
1	Male	75	5	2.7 **×** 23	Second	62	None	3
2	Male	79	4	2.7 **×** 25	Second	60	None	5
3	Male	80	5	3.0 **×** 25	Second	55	None	3
4	Male	85	5	2.5 **×** 23.5	Second	65	None	4
5	Female	72	4	3.0 **×** 24.5	Second	62	None	3
6	Male	80	4	3.0 **×** 25	Third	70	None	3
7	Male	82	5	2.8 **×** 23.5	Second	55	Hematoma	7

Abbreviations: IMAP, internal mammary artery perforator; L, length; W, width.

## Discussion

Reconstruction of defects following oncologic surgery in the head and neck, particularly in patients with prior radiation therapy, poses a significant challenge in plastic and reconstructive surgery. Complex cases in which neck vessels lack adequate coverage require reconstruction with vascularized tissue; in such scenarios, the use of vascularized flaps is recommended. 9

Propeller flaps have been used extensively to reconstruct various areas, including the lower and upper extremities, head, neck, chest, and face. 11 Additionally, the combination of the propeller flap technique with an IMAP flap is a recent option for breast, chest, and neck reconstruction. 12

The IMAP flap of the second intercostal space is considered safe due to the dominant perforator consistently arising from this space, and the average length of the enhanced vascular pedicle is 92 mm, with larger perforator dimensions and higher perfusion pressure. 13

In our study, only one patient (14.28%) treated with the IMAP flap experienced complications within the first 48 hours postintervention, with no cases of fistulas, infections, or long-term (3 months) complications. Other studies using radial and ALT flaps have reported fistula rates of 56.6% and 30.2%, respectively. 14

The IMAP flap technique significantly reduced surgical time for flap elevation to 61 minutes compared to free flap procedures, which can take up to 9 hours. 15 Also, IMAP flaps do not involve muscle groups, thereby avoiding functional impairment as in the ALT flaps that can affect the vastus lateralis muscle, potentially compromising knee and hip mobility. 16

In our study, six of seven surgeries were performed using the second intercostal space. The IMAP flap perforator course is very consistent, as its anatomy is very reliable and can be followed without an intraoperative ultrasound. 17 To ensure the surgical procedures of the study, we use Doppler ultrasound to locate perforator arteries and identify anatomical variations to optimize the precision of flap planning and improve surgical outcomes. 18 Nevertheless, because the flap is designed narrowly, vascular structures could deviate from the design, leading to partial flap loss, particularly in the tip area. In this case series, we did not observe evidence of partial or complete necrosis; however, it is important to consider that this can occur inherently in some cases. Moreover, flap perfusion evaluation with indocyanine green (ICG) angiography would increase its efficacy and safety.

The IMAP flap is thin and closely resembles the neck's native tissue, yielding superior aesthetic outcomes. 12 In cases where there are large defects or other types, such as cutaneous or tracheostomal, other flaps, such as the radial forearm flap, can be used to provide coverage.

In our study, defect sizes ranged between 4 and 5 cm, while the IMAP flap designs were planned between 2.5 cm wide and 23 to 25 cm long, providing complete coverage of the defects to be covered. IMAP flap of the second intercostal space supplies a vascular territory of 138 cm ^2^ , which is the largest detected skin dimension of IMAP. 17 As described previously, perforators with a bigger vascular territory have a higher freedom in flap orientation. In our cases, the IMAP flap had a 90- and 180-degree rotation angle. 19

We observed that our design of the IMAP flap for TL is efficient in reconstructive procedures among irradiated patients. Previous reports used similar techniques in five cases to reconstruct tracheostomes, suspend the trachea, and resurface the lower neck. 12 Flaps, such as the ALT, latissimus dorsi (LD), deep inferior epigastric perforator (DIEP), transverse rectus artery myocutaneous (TRAM), superficial cervical artery, and free flaps have been successfully used for reconstruction in irradiated patients. 20 However, fat necrosis and major infections are associated with the TRAM flap for breast reconstruction in irradiated patients, 21 while free, LD, and DIEP flaps are considered safe options in the reconstruction of radiation-induced chronic ulcers. 22

### Conclusion

The IMAP flap, which is the narrow and elongated flap design reported in this case series, is a safe and effective option for reconstructing laryngeal defects, even in patients with a history of laryngeal cancer treated with radiation therapy. The IMAP flap offers shorter surgical time for flap elevation and lower morbidity. The use of Doppler ultrasound is recommended, but not mandatory, for flap design, perforator selection, and surgical planning to optimize outcomes. However, further prospective studies with long-term follow-up are needed to assess its performance in irradiated tissues and compare its functional outcomes with other reconstructive techniques.
